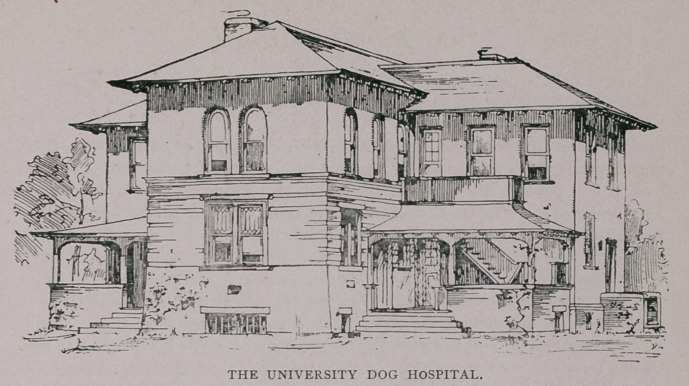# University of Pennsylvania—Hospital for Dogs*The authorities of the University of Pennsylvania, in a previous article upon the canine hospital and in this communication, while giving credit to various persons for its conception and completion, entirely ignore the fact that this entire scheme, except the exterior design of the building (which is not in conformity with the other buildings of the Veterinary Department, and should have been), was in the original plans prepared by Dr. Huidekoper when he organized and directed the foundation of the school, and the plans are to be found in his original communications to the Trustees.

**Published:** 1893-01

**Authors:** 


THE UNIVERSITY DOG HOSPITAL.

### UNIVERSITY OF PENNSYLVANIA—VETERINARY DEPARTMENT.*

### HOSPITAL FOR DOGS.

The recently completed hospital for dogs at the University of Pennsylvania was formally opened on Saturday afternoon with a simple service held in the University chapel. Dr. John Marshall, Dean of the Veterinary Department, presided. Provost Pepper was unable to be present through illness. The keys of the new hospital were turned over by Joseph E. Gillingham, President of its Board of Managers, to the trustees of the University, for whom they were received by H. H. Furness, LL.D.

Mr. Gillingham's address was a short one. He made an earnest appeal for funds to enable the department to carry on its good work, and said that during the year 1892, ending on August 31, there had been 1825 domestic animals treated. Dr. Furness, in accepting the keys on behalf of the Board, said:

Whatever else our fair building, with all its beneficent appliances, may effect, we shall all agree, I think, that in this town hereafter the language of anger or contempt has lost a favorite phrase. Who, hereafter, with any shadow of regret or malevolence, can say that a fellow-creature has gone to the dogs? A ban has been converted into a blessing; an abode of wrath into a delectable retreat.

Moreover, the kindness that is here shown is of the purest, untainted by any expectation of reward, or even of any audible expression of thanks. Time and money and skill have been, and are to be, here expended from the most unalloyed, the sublimated, motives of gentleness and tender mercy. There is no hope for us of any good round legacy in the wills of any of our patients; their good will here and now are our only guerdon. Neither can we expect that our sombre labors will be entwined by any mirth from our convalescents; there will be no sound of laughter, not even the merry tale of a wag; our highest hopes are bounded by the wag of a tail. And we have other limitations. If we here foster the nine muses they must perforce be canine; moreover we must blink the temperance question altogether, and at our threshold Prohibition must halt its domineering foot. It cannot be helped. It is inevitable. Wherever there are dogs there must be lickers—

* The authorities of the University of Pennsylvania, in a previous article upon the canine hospital and in this communication, while giving credit to various persons for its conception and completion, entirely ignore the fact that this entire scheme, except the exterior design of the building (which is not in conformity with the other buildings of the Veterinary Department, and should have been), was in the original plans prepared by Dr. Huidekoper when he organized and directed the foundation of the school, and the plans are to be found in his original communications to the Trustees.

and there will be whines, and yet I'll warrant you we'll never have a case of inebriation—but if we should, we'll not throw physics to the dogs; we'll give it to them gently with a spoon.

We accord high praise, and justly, to our own beloved physicians, who, from the symptoms which we describe to them, can diagnose our ailments, and we admire their skill. But how is it where the patient can give no atom of aid by describing his symptoms, and where not even an organization common to both patient and physician can be called on for help? It would be difficult to decide whether powers of observation were not demanded in the treatment of dumb animals keener and closer even than that of man.

In the name and on behalf of the Trustees of the University of Pennsylvania I accept this gift, and am happy in accepting it from hands the most worthy of all others to bestow it. And this symbolic key unlocks other doors besides those of the Canine Infirmary. At its turning the doors of memory unfold and the shadowy past again becomes real before us; and again we see the happy hour when the Veterinary Department, whereof this Canine Infirmary is a ward, was founded by the late J. B. Lippincott. So quietly, so unostentatiously was the large sum of $20,000 given that we, his co-trustees, were scarcely aware of the gift until its presence was revealed in the treasurer's columns. As far as any blare of self-laudatory trumpets was concerned, the gift was as dumb as any of the animals whom it has since blessed. The school, thus called into existence, has been accepted by the family of Mr. Lippincott as a sacred legacy, and a munificent contribution is annually bestowed on it by them. Thrice happy they who can wreath a father's monument with fresh undying garlands!

There is a second munificent contributor to whom the Veterinary School and Hospital owe their foundation and continued support, and as I do not see him here among you, I do not hesitate to name him and tell you that it is Mr. Joseph E. Gillingham, the present President of the Board of Managers of the Veterinary Hospital, who, subsequent to the gift of Mr. Lippincott, and equally as unostentatiously, presented the school with ten thousand dollars, and whose sound, mature wisdom, extended knowledge and unflagging zeal supplement his open hand in all the best interests of the department.

At the conclusion of Dr. Furness's address the invited guests, among whom were J. B. Lippincott, the Rev. Charles Dana Boardman, D.D., Frank Miles Day, Richard Wood, Dr. Raynor, Dr. Pearson, Dr. S. J. Harger, and Charles E. Dana, were shown through the new hospital.

The new hospital is said to be very complete and efficient and the first in this country. It stands at the west of the Veterinary Hospital. The exterior is of Roman brick, with pebble dashing and redstone trimming, and a roof of unfading green slate, the whole presenting a most unique appearance. The building covers an area of 65 by 50 feet. It is two stories high, with a basement, in which is situated the kitchen for cooking the food for the canines. The first floor contains a clinic room, which opens into a larger room, in which will be treated animals affected with non-contagious diseases. On opposite sides of this are the wards for distemper and mange.

The second story is divided into four rooms, which will probably be used as laboratories for original research in veterinary medicine. All the rooms are fitted up with most complete heating and ventilating appliances. They will be lighted by electricity, and electric fans will keep the air at a suitable temperature. Baths for the dogs are provided, and the animals themselves will be kept in iron cages, which will be placed on wheels to permit of easy moving.

## Figures and Tables

**Figure f1:**